# Natural exposure of bats in Grenada to rabies virus

**DOI:** 10.1080/20008686.2017.1332935

**Published:** 2017-06-19

**Authors:** Ulrike Zieger, Sonia Cheetham, Sharlene E. Santana, Leith Leiser-Miller, Vanessa Matthew-Belmar, Hooman Goharriz, Anthony R. Fooks

**Affiliations:** ^a^ Department of Anatomy, Physiology and Toxicology, School of Veterinary Medicine, Wildlife Conservation Medicine Program, St George’s University, St George’s, Grenada; ^b^ Pathobiology Department, School of Veterinary Medicine, St George’s University, St George’s, Grenada; ^c^ Department of Biology and Burke Museum of Natural History and Culture, University of Washington, Seattle, WA, USA; ^d^ Wildlife Zoonoses and Vector-Borne Diseases Research Group, Animal and Plant Health Agency, Weybridge, UK; ^e^ Institute for Infection and Immunity, St George’s Hospital Medical School, University of London, London, UK

**Keywords:** *Artibeus jamaicensis*, bats, Grenada, serology, rabies, virus

## Abstract

**Introduction**: Grenada is a rabies endemic country, where terrestrial rabies is maintained in the small Indian mongoose (*Herpestes auropunctatus*). The role of bats in the epidemiology of rabies in Grenada is unknown. A 1974 report described one rabies virus positive Jamaican fruit bat (*Artibeus jamaicensis*), and a high seroprevalence in this species. In the current study, the natural exposure to rabies virus in Grenadian bats was re-evaluated. It is postulated that bats serve as a natural rabies reservoir, probably circulating a bat-specific rabies virus variant.

**Material and methods**: Bats were trapped in 2015 in all six parishes of Grenada using mist- and hand nets. For the detection of rabies virus in brain tissue, the direct fluorescent antibody test (dFAT) and the reverse transcription polymerase chain reaction (RT-PCR) were used. Serum neutralizing antibodies were determined using the fluorescent antibody virus neutralization test (FAVN).

**Results and discussion**: Brain tissue and sera from 111 insectivorous and frugivorous bats belonging to four species were tested (52 *Artibeus jamaicensis*, two *Artibeus lituratus*, 33 *Glossophaga longirostris*, 24 *Molossus molossus*). Rabies virus antigen and genomic RNA were not detected in brain tissues. Rabies virus neutralizing antibodies were detected in the sera of eight *A. jamaicensis* in four of the six parishes. Bats in Grenada continue to show natural exposure to rabies virus. As rabies virus was not isolated in this study, serology alone is not sufficient to determine the strain of rabies virus circulating in *A. jamaicensis* bats in Grenada.

**Conclusion**: *Artibeus jamaicensis* appears to play a role as a reservoir bat species, which is of public health concern in Grenada. Dispersion of bats to neighboring islands is possible and serological bat surveys should be initiated in these neighboring states, especially in those areas that are free of rabies in terrestrial mammals.

## Introduction

Rabies lyssavirus (RABLV) infection is enzootic in bats in North America, where the virus has been detected in most known bat species.[1] Of the 14 recognized virus species in the *Lyssavirus* genus that can cause rabies, rabies virus (RABV) is the only one that circulates in the New World.[2] Many RABV variants exist, and are mostly species-specific.[3] However, cross-species transmission occurs among bats species and between bats and terrestrial mammals, and bat-associated rabies is now the main source of human rabies in the USA and Canada since canine rabies is largely controlled.[4]

In Latin America and the Caribbean, rabies surveys and control measures have focused on terrestrial RABV reservoirs, and on the hematophagous or vampire bat species where they exist. Information on other bat species as potential rabies reservoirs in the Neotropics is scarce. This is also true for Grenada, one of four Caribbean islands where rabies is endemic, and is maintained in the small Indian mongoose (*Herpestes auropuntatus*) with spillover into domestic animals. The RABV variant that circulates in Grenadian terrestrial mammals has recently been described.[5] The role of bats, however, in the rabies epidemiology in Grenada is unknown. One report exists dating back to the 1970s, in which RABV was detected in the brain of one Jamaican fruit bat (*Artibeus jamaicensis*).[6] The same study detected rabies virus neutralizing antibodies in six bat species, with the highest seroprevalence in *A. jamaicensis.*

In the current study, the natural exposure to RABV in Grenadian bats was re-evaluated. The working hypothesis is that Grenadian bats serve as a natural rabies reservoir, with circulation of a bat-specific variant of rabies virus. Implications for the spread of bat rabies virus to neighboring islands and the public health consequences are discussed.

## Material and methods

### Ethics statement

St George’s University (Grenada) Institutional Animal Care and Use Committee (IACUC) approved all animal work on 3 December 2014 under IACUC # 14008.

### Sample collection

*Artibeus jamaicensis, Artibeus lituratus* and *Glossophaga longirostris* were collected during the day using hand nets at their roosting sites (one cave and several abandoned buildings) and at night using mist nets in forested and urban areas. Because of *Molossus molossus*’ flight behavior (high flight), mist nets were placed in front of their roosts to capture emerging bats at dusk. Bats were placed in individual cloth bags, transported to the lab and euthanized by isoflurane overdose. All bats looked healthy at the time of collection. Blood samples were taken immediately after euthanasia via cardiac puncture. Sera were heat inactivated for 30 min at 56°C and stored at −20°C until processing. Brains were removed and stored dry at −20°C until processing.

### RABV antigen detection

Rabies was diagnosed by the detection of viral antigen in acetone fixed brain tissue using the direct fluorescent antibody test (dFAT).[7] A cocktail of three fluorescein-labeled monoclonal antibodies directed against the rabies nucleocapsid (N) protein (Light Diagnostics^TM^ Rabies DFA reagent, Millipore, Livingston, UK) was employed following supplier’s instructions.

### RNA extraction and RT-PCR

Total RNA was extracted from 20 to 30 mg brain tissue after tissue lysis in a bead-beater (Mini Beatbeater^TM^ Biospec Products, Bartlesville, OK, USA) and using RNeasy Mini Kit spin columns (Qiagen GmbH, Hilden, Germany) following the manufacturer’s instructions. RABV RNA was detected via RT-PCR as recently described.[5] The primers used for amplification targeted a 110 bp fragment of the highly conserved region of the nucleoprotein (N)-gene: JW 12 (5ʹ-ATGTAACACCYCTACAATG-3ʹ) and N 165-146 (5ʹ-GCAGGGTAYTTRTACTCATA-3ʹ).

### Serology

In summary, Grenadian bats are exposed to rabies virus (RABV) and may the presence of rabies virus neutralizing antibodies was measured in heat inactivated sera using the fluorescent antibody virus neutralization (FAVN) test with a fixed quantity of rabies virus (challenge virus standard; CVS-11) as previously described.[8] Titers are expressed in IU (international units) per mL by comparison to a standard serum and using the standard threshold of greater than or equal to 0.5 IU/mL to determine seropositivity.

### Statistics

Confidence limits at 95% for the reported seroprevalence rates were calculated using MedCalc statistical software (MedCalc bvba, Ostend, Belgium).

## Results

**Figure 1.**
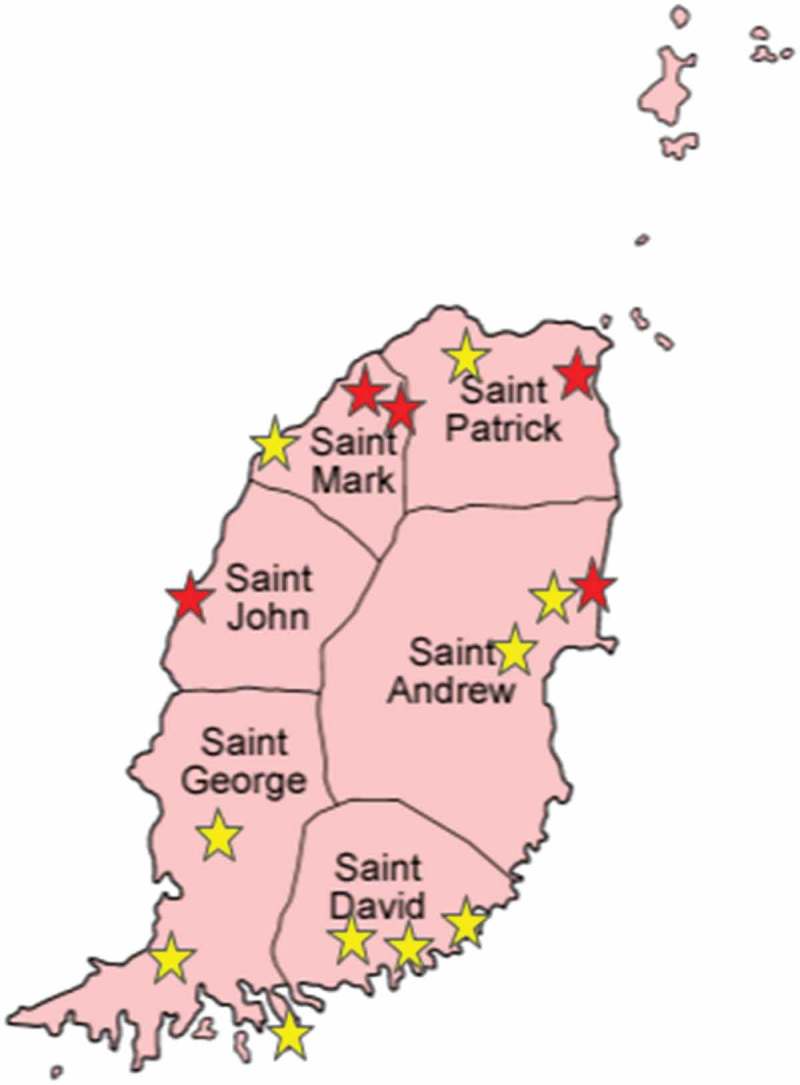
Map of Grenada and its six parishes, showing the 15 bat trapping locations; red stars indicate locations where seropositive bats were found.

A total of 111 bats belonging to four species were examined: 52 *A. jamaicensis*, two *A. lituratus*, 33 *M. molossus*, and 24 *G. longirostris*. Their trapping locations are shown in Figure 1, and bat species and numbers per parish are shown in Table 1.

**Table 1.** Bat species and bat numbers per parish testing positive for rabies virus antigen (dFAT), rabies virus genomic RNA (RT-PCR) and rabies virus neutralizing antibodies (FAVN).

	dFAT	RT-PCR	Serology (FAVN)						
	Total no.	Total no.	Total no.	St George	St David	St John	St Mark	St Patrick	St Andrew
*A. jamaicensis*	0/52	0/52	**8/52 (15.4%)**	0/8	0/7	3/19	2/11	1/3	2/4
*M. molossus*	0/33	0/33	**0/33**	0/3	0/8	0/0	0/4	0/12	0/6
*G. longirostris*	0/24	0/24	**0/24**	0/5	0/5	0/0	0/3	0/4	0/7
*A. lituratus*	0/2	0/2	**0/2**	0/1	0/0	0/0	0/1	0/0	0/0
Total no.	**0/111**	**0/111**	**8/111 (7.2%)**	**0/17**	**0/20**	**3/19**	**2/19**	**1/19**	**2/17**

dFAT (direct fluorescent antibody test); RT-PCR (reverse transcription polymerase chain reaction).

RABV antigen or viral RNA was not detected in any of the 111 brain samples tested by dFAT and RT-PCR (Table 1). RABV neutralizing antibodies were detected in the sera of eight of 111 bats (Table 1) (7.2%; 95% CI 3.4–14.1%), with titers ranging from 0.66 to 3.42 IU/mL. All eight seropositive bats were adult *A. jamaicensis* (eight of 52 or 15.4%; 95% CI 7.3–28.6%); three were females and five males. Seropositive bats were trapped in four of Grenada’s six parishes (Figure 1).

## Discussion

In Grenada, terrestrial and bat rabies co-exist. The results from our study for *Artibeus jamaicensis* are comparable to those of Price and Everard of 1977.[6] These authors reported a seroprevalence of 40.5% (17/42) in all *A. jamaicensis*, respectively 31.4% (11/35) when non-flying infants were excluded. We showed that 15.4% (8/52) of *A. jamaicensis* were seropositive, all of which were flying juveniles or adults. There were no seropositive animals in the other three species trapped in our study, which may be due to an insufficient sample size: in the previous study,[6] the seroprevalence of *M. molossus* and *G. longirostris* was below 2%, and *A. lituratus* had not been captured.

The differences in seroprevalence rates between the previous and the current study are not surprising. A 12-year longitudinal study has demonstrated significant inter-annual variations and cyclic lyssavirus infection in greater mouse-eared bats (*Myotis myotis*), which ‘occurred with periodic oscillations in the number of susceptible, immune and infected bats’.[9, p. 5] The maintenance of a virus in a bat population depends upon numerous environmental and anthropological factors as well as on the mode of virus transmission, infectious period, and other epidemiological parameters.[10,11] Host population viability is also highly dependent on these external factors and will have a clear effect on infection dynamics. In addition, seasonal bat behavior, especially the bat birth pulse, will result in changes in population size that will clearly affect the rate of contacts among bats.[12] The persistence of antibodies to RABV in *A. jamaicensis* bats from Grenada indicates that these factors may explain differences in seroprevalence rates in the same species of bats from different years. It could be assumed that if the population size decreases below a minimum threshold that the virus would be expected to fade out. These seroprevalence data, however, suggest that the virus is being maintained within *A. jamaicensis* bats in Grenada and that the virus endemically circulates at a low and fluctuating level. The question of how infection is maintained in the absence of significant observed disease or mortality needs to be resolved to fully understand the public health implications.

Our study confirms that at least one bat species in Grenada, the Jamaican fruit bat (*A. jamaicensis*), continues to show natural exposure to RABV. There was no detection of RABV in the brains of our bats, indicating that they did not suffer from clinical rabies at the time of capture. This is not surprising, as all bats were trapped during flight or while roosting, which indicates that they were probably healthy at the time of sampling. So far, RABV is the only member of the *Lyssavirus* genus that has been described in the New World.[2,13] Because classical RABV shares cross-neutralizing antibodies with the other members of the *Lyssavirus* genus within phylogroup I,[14] it is not possible to conclude that it is RABV which circulates in Grenadian bats and not another or a new closely related virus in this phylogroup. The search to isolate and characterize a virus is underway.

RABV is believed to have evolved in bats with subsequent spill-over into terrestrial mammals.[15] Many reports describe high prevalence rates of RABV neutralizing antibodies in apparently healthy bats.[16,17] Several explanations are possible: perhaps bats are exposed to a RABV variant with low pathogenicity in this species, develop clinical signs that result in an abortive infection and the bats do not succumb to the disease. Alternatively, bats could be exposed to low doses of RABV, which allow an immune response to occur, resulting in a sterilizing immunity, but fail to cause disease. Also, juvenile bats could be exposed to RABV while still under passive immune protection and develop a ‘booster’ immune response after repeated exposure. Experimental infection studies in bats have shown that RABV neutralizing antibodies are short-lived,[16] e.g. titers in big brown bats (*Eptesicus fuscus*) fell below detectable levels 140 days after infection.[18] The actual rate of natural exposure to RABV must therefore be considerably higher than is reported in active surveillance studies.[17] On the other hand, seroprevalence data from naturally infected insectivorous bats from Europe suggest that bats can maintain their antibody titer for between two and five years.[19] It must also be stressed that the presence of RABV neutralizing antibodies does not necessarily protect a bat from developing rabies, neither does their absence indicate that a bat will succumb to rabies after exposure.[20] Other host protective mechanisms, such as the innate immune system or other antibody types, certainly contribute to a bat’s immune status.[21,22]

While seroprevalence rates reported elsewhere are generally high, relatively few rabid bats are reported and RABV detection rates in bat population studies are typically around or below 1% depending on sampling criteria.[23] There is no evidence that bats survive once clinical signs of rabies develop, neither is there conclusive evidence that a true carrier state exists.[24] How RABV is maintained so successfully in apparently healthy populations is still unknown, but must involve efficient transmission to achieve high seroprevalence rates, while still allowing sufficient time for infected bats to shed RABV in their saliva during clinical rabies. Little is known about transmission routes in bats, but these may involve bite or scratch exposure during attacks or grooming, or aerosol formation during vocalization or echolocation.[24] In a recent study, little brown bats (*Myotis lucifugus*) were inoculated intramuscularly, mimicking bite exposure. More than half of the infected bats succumbed to rabies, but their saliva was not infectious.[25] Following subcutaneous inoculation, such as could be expected to occur during grooming, few bats developed rabies, and these showed extended incubation times, shedding virus in their saliva for up to 18 days prior to developing clinical signs.[25] In colonial bat species that engage in extensive grooming behavior, this might be an important viral adaptation to maintain its presence in a host. It has been postulated that large bat population sizes and crowded roosting conditions facilitate intra- and interspecific viral transmission.[26] In Grenada, *A. jamaicensis* appears to form the largest colonies, with several hundred individuals. This might explain why the current and the previous study in Grenada [6] observed high seroprevalence rates only in this species. However, quantitative information concerning this bat’s colony sizes and roosting behavior in Grenada is missing.

It is unknown whether transmission from Grenadian bats to terrestrial mammals or humans has ever occurred. A recent study describing the RABV variant that circulates in the Grenadian small Indian mongoose with spillover into domestic animals has shown that a monophyletic clade exists, distinct from the usual bat-associated variants found elsewhere.[5] This does not exclude the possibility of transmission from bats to other mammals, but makes it less likely. However, it is interesting to note that for the last human rabies case that was reported in Grenada in 1970, no known bite exposure had occurred.[27] This is a typical history in bat rabies cases. Generally, RABV transmission from bats to terrestrial mammals tends to be a dead-end for the virus.[3] Yet, there are two reports which provide evidence that bat-associated RABV seems capable of establishing itself in a terrestrial host with subsequent rabies outbreaks in the new host population: in 1993 in foxes in Prince Edward Island, Canada [28] and in 2001 in striped skunks in Arizona, USA.[29]

The consistent finding of natural exposure to RABV in bats in Grenada remains a public health concern. Anyone exposed to a bat in Grenada, especially by being bitten, should seek immediate medical assistance. In most circumstances, especially when the bat is not available for testing, a full course of post-exposure prophylaxis should be administered to the patient as a precaution and following WHO guidelines.[30] This is also of relevance for neighboring islands. To our knowledge no studies have been conducted on the other Lesser Antillean islands that are considered rabies-free. Although bat migration patterns within the region are largely unknown, phylogenetic analysis of bats in the Lesser Antilles showed that the genetic structure of *A. jamaicensis* populations is not monophyletic within islands. This indicates that these bats disperse among islands, perhaps facilitated by heavy storms and hurricanes.[31]

In summary, Grenadian bats are exposed to rabies lyssavirus (RABLV) and may constitute a natural rabies virus reservoir, posing a potential threat to other mammalian species in Grenada and neighboring islands. It would be prudent to initiate active surveillance through serological bat surveys in neighboring states, even if these are considered rabies-free. Passive and disease surveillance studies are underway to determine the rabies lyssavirus variant circulating in Grenadian bats. These include testing clinically sick or dead bats, which is likely to be more successful in isolating the virus.
